# In vivo investigation of diode laser application on red complex bacteria in non-surgical periodontal therapy: a split-mouth randomised control trial

**DOI:** 10.1038/s41598-020-78435-7

**Published:** 2020-12-04

**Authors:** Suné Mulder-van Staden, Haly Holmes, Jos Hille

**Affiliations:** 1 Faculty of Dentistry, Oral Medicine and Periodontics, University of the Western Cape, Fransie van Zijl drive, Cape Town, 7505 South Africa; 2 Department of Oral and Maxillofacial Pathology, Faculty of Dentistry, Oral and Maxillofacial Pathology, University of the Western Cape, Cape Town, South Africa; 3 National Health Laboratory Service (NHLS), Tygerberg Academic Laboratories, Tygerberg Hospital, Cape Town, 7505 South Africa

**Keywords:** Microbiology, Diseases, Health care, Medical research, Signs and symptoms

## Abstract

Assessment of the efficacy of a single 810 nm diode laser application as an adjunctive treatment modality during the first intervention of non-surgical periodontal therapy (NPT). 25 patients diagnosed with chronic periodontitis underwent a split-mouth randomised control trial. The periodontal pockets of the test quadrants were treated with an 810 nm diode laser as an adjunct to NPT (Picasso GaAlAs; AMD Lasers). The laser was set at 1.0 W continuous wave, 400 µm tip, 796 W/cm^2^ peak power density and a 32 J/cm^2^ energy density. Therapeutic outcomes were evaluated based on the clinical parameters, which included probing pocket depth, recession, clinical attachment level, full mouth plaque score, full mouth bleeding on probing and tooth mobility. The baseline bacterial collection was completed from the periodontal pockets and then re-evaluated at 6 weeks. Clinical parameters demonstrated no statistical difference, with the exception of a statistically significant (*P* < 0.05) reduction in bleeding on probing for the test side. The test side resulted in a statistical increase of *Capnocytophaga* *species* and *Treponema* *denticola*. The single application of the diode laser did not significantly improve the bacterial nor the clinical parameters in patients with chronic periodontitis.

Trial registration number: PACTR201909915338276.

## Introduction

Periodontal disease is a multifactorial condition affecting most populations worldwide. It is characterized by tissue destruction and disease progression occurring as a result of complex interactions between micro-organisms, environmental factors and the host tissues^1,2^.

The red complex bacteria namely *Porphyromonas* *gingivalis* *(P.g.),* *Treponema* *denticola* *(T.d.)* and *Tannerella* *forsythia* *(T.f.*) have been demonstrated to be the predominant microbial species involved in periodontal disease progression, deeper probing pocket depths, bleeding and significant periodontal tissue destruction^3^. Numerous studies assessing the management of periodontitis have correlated successful periodontal management with elimination or suppression of these virulent red complex bacteria from the periodontal pockets^4^. However, accomplishing a reduction in the numbers of these destructive red complex bacteria, often remains an obstacle in the management of periodontal disease.

The aim of periodontal therapy is the elimination of supragingival and subgingival biofilms^5^. Studies have demonstrated that disruption of the bacterial biofilm is paramount to the effective management of periodontitis^6^. Non-surgical periodontal therapy (NPT—i.e. scaling, root planing, polishing) are the fundamentals of periodontal disease management^7^. However, NPT has its limitations in the complete removal of periodontal pathogens and is prone to both clinical and microbiological relapse^1,8^. These limitations may be attributed to several factors—such as complex tooth anatomy, presence of intrabony defects, limited access associated with the size of instrumentation and invasion of periodontal pathogens into the surrounding soft tissues^1,5^. NPT may also only result in a temporary shift in the composition of the subgingival microflora with persistence of periodontopathic bacteria, resulting in a potential for recolonization of treated sites^5^. The lack of long-term successful treatment outcomes of NPT in some chronic periodontitis cases has emphasized the need for the identification of adjunctive management modalities^9^.

Dental lasers reportedly have numerous applications in periodontal therapy, such as surgical procedures of soft tissue and osseous structures, non-surgical treatments such as pathogen reduction, removal of surface accretions and photobiomodulation^10^. The laser functions on the principle of energy transmission to the area of application. Tissue interactions that can occur during laser application include reflection, transmission, scattering and absorption. Transmission occurs by virtue of the crevicular fluid in the periodontal pocket, as it does not absorb the laser light. In biologic tissues, scatter of the absorbed laser energy occurs upon deep tissue penetration. This absorbed energy is converted to heat and therefore increases the tissue temperature^11^.

Various commercially available lasers have been investigated in periodontal treatment, each with a unique range of wavelengths that can be adjusted according to the procedure requirements. Wavelengths differ dependent on the type of laser utilised (such as the CO_2_ laser, Er:YAG laser, Nd:YAG laser and the gallium–aluminium–arsenium (GaAlAs) diode laser) and these wavelengths range between 635 to 10,600 nm^12–14^.

Laser energy absorption by the target tissues depend on the laser wavelengths. Lasers that emit energy in the intermediate infra-red spectrum, such as CO_2_ (10,600 nm) and the Erbium lasers (Er:YAG 2940 nm; Er:YSGG 2780 nm) are absorbed primarily by water (and secondarily by apatite) and operate chiefly through vaporization. The CO_2_ laser is reported to be effective for soft tissue surgery related to haemostatic and bactericidal effects. CO_2_ laser energy is absorbed by inorganic compounds, such as dental hard tissue and bone. Thus the disadvantage of CO_2_ lasers is potential thermal damage of bone and dental hard tissue. This thermal damage can significantly alter the root surface and surrounding bone, thus hindering periodontal re-attachment post-operatively. The use of CO_2_ laser for periodontal treatment is limited and the existing literature is not sufficient to suggest specific treatment recommendations^14^. Comparison of the application of the Er:YAG laser to hand instrumentation, sonic and ultrasonic devices was conducted in patients with chronic periodontitis. At the 3 month post-operative micro-biological assessment Er:YAG and sonic devices failed to reduce *A.a*. The Er:YAG laser was demonstrated to be equivalent to hand instrumentation, sonic and ultrasonic devices in the reduction of the levels of *P.g.*, *P.i.*, *T.f.* and *T.d.*^15^. Er:YAG lasers induce photo-thermal evaporation of water contained in mineralised tissue, resulting in ablation of a thin layer of the targeted tissue. Thus a potential disadvantage of the Er:YAG laser is the undesired removal of cementum and dentin^14^. Lasers emitting in the near infra-red spectrum, such as Nd:YAG (1064 nm) and diode lasers (655–980 nm) are absorbed by tissue macromolecules and pigments and operate chiefly by coagulation and carbonization^16,17^. The advantageous potential bactericidal properties of these near infra-red spectrum lasers have been described in the literature^18^. The bactericidal mechanism is hypothesized to be associated with laser energy which is selectively absorbed by dark pigments (photodynamic therapy on the dark pigment), such as melanin and haemoglobin^18^. In inflamed periodontal pockets, the increased blood supply due to erythema and inflammation causes the laser light to be absorbed by the haemoglobin, which is elevated in the inflamed pockets^19^. Chromophores (associated with periodontal inflammation) will result in greater absorption of laser energy in the inflamed tissue compared to healthy tissue^19^. Nd:YAG lasers have been reported to induce undesirable changes on root surfaces, such as charring and melting^20^. To prevent severe thermal damage to dental pulp and root surfaces, narrow setting parameters are recommended (1.5–3 W (60–150 mJ/pulse at 10–20 Hz). This limits the clinical application of Nd:YAG lasers and accounts for the controversial reports and overall limited clinical evidence regarding the actual efficacy in periodontology^14^.

The diode laser is a semiconductor laser that generally includes a combination of gallium (Ga), arsenide (As), and other elements such as aluminum (Al), and indium (In) to convert electrical energy to light energy. The operating wavelengths of diode lasers (besides thermal transfer) do not interact with mineralized tissues, allowing safe usage for procedures such as sulcular debridement, achieving soft tissue ablation, haemostasis and sterilization even in close proximity to dental roots in periodontal pockets^17,19^. Theoretically, an initiated tip at the appropriate peak power should only affect and eliminate inflamed tissue, with a lesser effect on the healthy tissue in the periodontal pocket^19^. The red complex bacteria possess numerous virulence factors, such as the ability to infiltrate along the tissue capillaries and penetrate the periodontal pocket connective tissue to a depth of 500 µm, making their elimination potentially difficult^11^. Deep periodontal pockets also present with higher numbers of pigmented bacteria, thus allowing greater absorption of the diode laser energy^11^. It is thus assumed that the diode laser could be an adjunct in reducing the colony numbers of these pigmented, red complex bacteria^18^. The tissue effect of diode lasers is similar to the Nd:YAG, however, less negative thermal effects on the deeper tissue is reported^21^. Diode lasers can also penetrate 1–3 mm into the tissues^11^. This depth of penetration is influenced by many factors. These include the presence of chromophores (including haemoglobin and melanin pigments), inflammation and the power/energy settings of the laser. Diode laser application has also been shown to have an impact on non-pigmented bacteria by virtue of its thermal properties, resulting in a temperature increase within the periodontal pocket. The absorbed laser energy also stimulates the proliferation of endothelial cells, which promotes healing and local blood circulation by way of photobiomodulation^11^. This study and other authors^22–25^ selected the 810 nm diode laser based on the advantages of effective soft tissue penetration, an affinity for pigmented bacteria and inflamed periodontal tissue, without damaging tooth structure (below 1.5 W) in periodontal pockets.

The aim and rationale of this study was to evaluate and directly compare the efficacy of the first intervention of NPT alone (control side) with that of NPT including adjunctive single diode laser (810 nm) application (test side). The objectives were to compare the changes in both bacterial load of pathogens (bacterial parameters) and clinical parameters in the test and control groups. The null hypothesis (H_0_) is that the improvement of treatment outcomes (clinical/bacterial parameters) of quadrants treated with NPT with adjunctive laser therapy is not different to NPT alone. The alternative hypothesis (H_a_) is that the improvement of treatment outcomes (clinical/bacterial parameters) of quadrants treated with NPT with adjunctive laser therapy is different to NPT alone.

## Materials and methods

### Study design

A randomised control split-mouth trial was completed at the Tygerberg Oral Health Centre, at The University of the Western Cape, South Africa. The patients presented at the Oral Medicine and Periodontology Department. These patients provided informed consent. Treatment and recruitment procedures were in accordance with good clinical practice and the Declaration of Helsinki. The study protocol was approved by the UWC Senate Research Committee Ethical review board of the University of the Western Cape (Project Registration number 14/9/6) and a clinical trial registration number PACTR201909915338276 obtained at the PAN African Clinical Trials Registry 20/08/2019^26^.

### Study population

The study population had to have a diagnosis of untreated chronic periodontitis—according to the criteria of the American Academy of Periodontology Classification, 1999^27^. The study was conducted prior to the introduction of the 2017 Classification. The clinical features defining a diagnosis of chronic periodontitis included: Edema and tissue erythema, moderate accumulations of plaque and calculus, bleeding on probing (BOP), increased pocket depths (minimum probing pocket depth of 5 mm), radiographic evidence of bone loss (angular or horizontal), tooth mobility, varying degrees of clinical attachment loss (CAL), slow to moderate rate of progression and the amount of destruction consistent with the presence of local factors—such as plaque and calculus^27^.

Participants included in this study had to be over the age of 18 years and diagnosed with chronic periodontitis^27^ with a minimum of four teeth present per quadrant. The chronic periodontitis could have presented as localized (< 30% of sites affected) or generalized (> 30% of sites affected). The severity of the chronic periodontitis could have presented as mild (CAL = 1–2 mm), moderate (CAL = 3–4 mm) or severe (CAL > 5 mm)^4^. All the above-mentioned variants of chronic periodontitis were included in this study. Additionally, at least three teeth per quadrant had to present with periodontal probing pocket depths of 5 mm or more in any of the six-point probing areas namely mesio-buccal (MB), buccal (B), disto-buccal (DB), mesio-palatal (MP), palatal (P) and disto-palatal (DP).

Patients with underlying medical conditions such as pregnancy (or lactating) and those undergoing radiation or chemotherapy were excluded^23^. Patients with oral pigmentation of the attached gingiva in the study area of interest were also excluded from the study. Pigments (such as melanin) absorb laser energy at varying amounts^11^ which could potentially skew the results of the diode laser application. Smokers using more than 10 cigarettes per day^23^, patients that had taken antibiotics (in last 6 months) or had NPT in the previous 6 months were also excluded^25^.

### Study interventions

#### Bacterial collection and assessment of parameters protocol

The analysis of the collected bacteria included the following micro-organisms: *Aggregatibacter* *actinomycetemcomitans* *(A.a.);* *Porphyromonas* *gingivalis* *(P.g.)*; *Prevotella* *intermedia* *(P.i.)*; *Tannerella* *forsythia* *(T.f.)*; *Treponema* *denticola* *(T.d.)*; *Peptostreptococcus* *micros* *(P.m.)*; *Fusobacterium* *nucleatum* *(F.n.)*; *Campylobacter* *rectus* *(C.r.)*; *Eubacterium* *nodatum* *(E.n.)*; *Eikenella* *corrodens* *(E.c.)* and *Capnocytophaga* *species* *(C.s.).*

The full-mouth periodontal examination (FMPE) and the bacterial collection was performed prior to the commencement of the NPT and the assignment of test/control quadrants. Bacteria were collected from the subgingival aspect (with the sterile paper points provided) with a commercially available kit (Micro-IDent-11; Hain Lifescience, Nehren, Germany)^28^. The manufacturers’ instructions were followed with one PCR-test kit per side. Two paper points were utilized in the deepest periodontal pocket per tooth on the buccal (MB, B, DB) and palatal (MP, P, DP) aspects. The split-mouth design allowed the bacterial collection per side to be grouped (control quadrants as a pooled sample for the PCR-test kit and the test quadrants as a pooled sample for the PCR-test kit) to make an extract for the two polymerase chain reaction tests (PCR-tests) per appointment. Six weeks after the first intervention, the bacterial collection was repeated at the same baseline periodontal sites with two bacterial assessment kits (Micro-IDent-11; Hain Lifescience, Nehren, Germany), where one randomly selected side received only NPT (control side) and the other side received NPT plus the diode laser application (test side).

### Bacterial PCR analysis

#### DNA isolation

DNA isolation from the paper points: 400 μl 5% styrene divinylbenzene copolymer (Chelex 100; Bio-Rad, California, USA) in 10 mM tris(hydroxymethyl)aminomethane with a pH 8.5 were added to every sample. After centrifugation of the paper points, the samples were placed in an ultrasonic bath (Branson 5510 DTH; Branson Ultrasonics, Lawrenceville, USA) at 60 °C, for 15 min. Finally, the samples were incubated for 15 min in a 105 °C thermo-block. Following centrifugation, 5 μl was used for the polymerase chain reaction^28^.

#### PCR amplification

PCR amplification was carried out in a reaction volume of 50 µl, consisting of 5 μl of template DNA and 45 µl reaction mixture containing 35 μl primer nucleotide mix (Multiplex Micro-IDent kit; Hain Lifescience, Nehren, Germany), 5 µl of PCR buffer (10X PCR buffer; Qiagen, Hilden, Germany), 5 µl of 25 mM MgCl_2_ and 1 Unit of polymerase (Taq polymerase; Qiagen, Hilden, Germany). The amplification profile was one cycle at 95 °C, for 5 min; ten cycles at 95 °C, for 30 s and 58 °C, for 2 min; 20 cycles at 95 °C, for 25 s; 53 °C, for 40 s and 70 °C, for 40 s and one final cycle at 70 °C, for 8 min. For the *Peptostreptococcus* species until *Capnocytophaga*, a second primer nucleotide mix was used (Multiplex Micro-IDent-11 Plus kit; Hain Lifescience, Nehren, Germany)^28^.

#### Reverse hybridization

**Figure 1:**
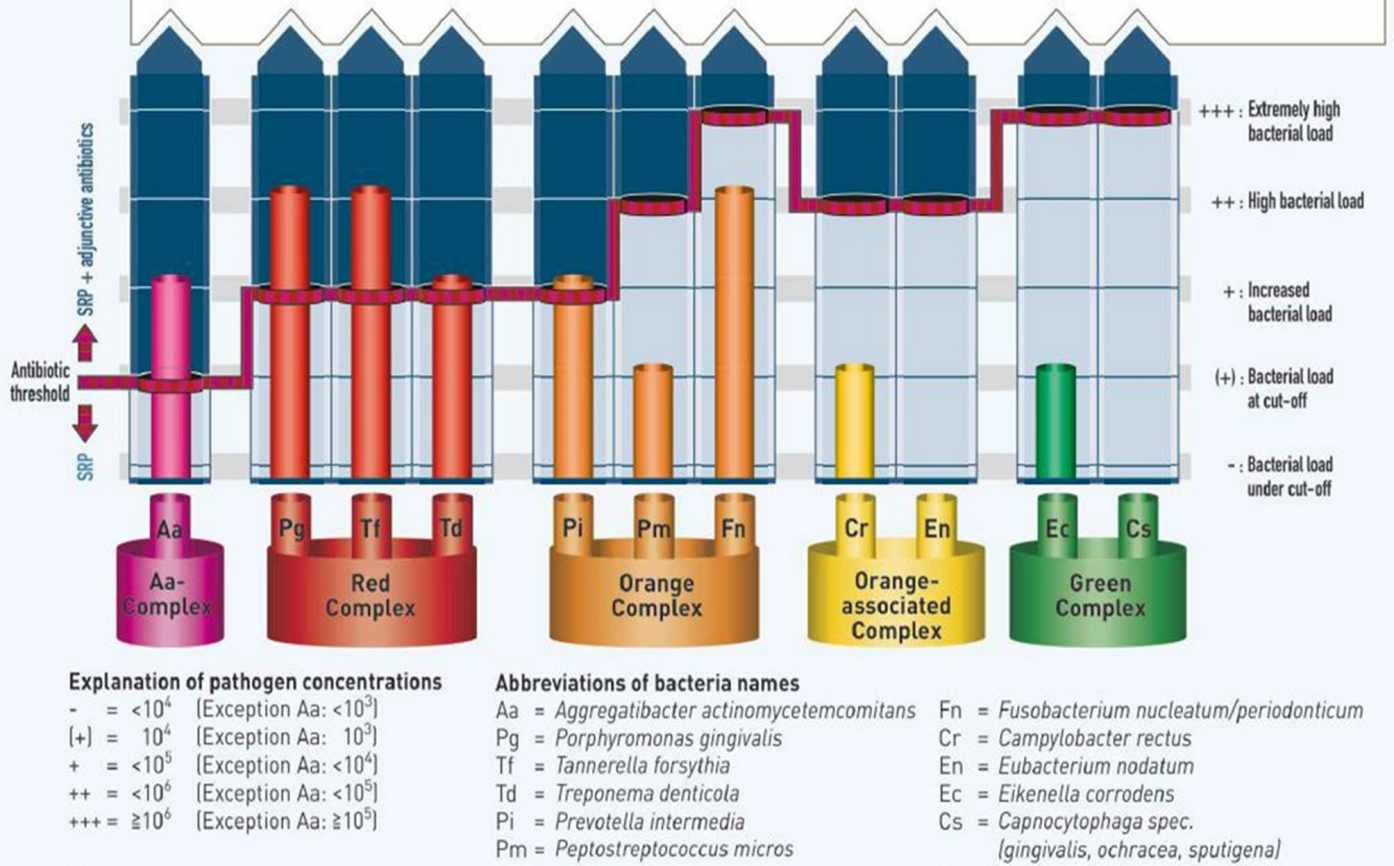
Illustration of pathogen concentrations from a bacterial report (*Printed* *with* *permission* *from* *Hain* *Lifescience,* *Nehren,* *Germany*)*.*

For the automatic executed hybridization, a thermal blot bioanalyzer (Apollo analyzer; Matec Medizintechnik, Münsingen, Germany) was used; 20 μl of the amplified sample is mixed with 20 μl of the denaturizing solution, for 5 min at room temperature (± 23 °C). One milliliter of pre-warmed hybridization buffer was added. The specific DNA probes for the reverse hybridization are fixed on a membrane strip. Under gentle shaking, the strip was incubated at 45 °C for 30 min. After aspiration of the hybridization buffer, 1 ml of stringent wash buffer was added and the strip was incubated at 45 °C for 15 min. The strip was washed for 1 min with the rinse solution at room temperature. The conjugate (streptavidin-conjugated alkaline phosphatase) was added, and the strip was incubated for 30 min at room temperature. After being washed twice for 1 min with water, the strip was dried between absorbing papers, and the results are evaluated, quantified and interpreted. Validated quantitative and qualitative test samples are used as control samples. Bacterial levels were expressed as genome equivalents (< 10^3^ = 0; 10^3^–10^4^ = 1; 10^4^–10^5^ = 2 and 10^5^–10^6^ = 3). The test has a detection limit of 10^3^ genome equivalents. The bacterial assessment kit (Micro-IDent-11; Hain Lifescience, Nehren, Germany) report of analysis visually illustrates the bacterial parameter sensitivity range^28^ (Fig. 1).

### Full-mouth periodontal examination (FMPE)^27^

#### Probing pocket depth (PPD)

PPD was measured to the nearest millimeter with a graduated periodontal probe (Maillefer; Sirona Dentsply, Charlotte, USA) from the gingival margin to the base of the clinical pocket^4^. Six sites MB, B, DB, MP, P and DP per tooth were assessed.

#### Recession (REC)

Recession (REC) was measured with a graduated periodontal probe (Maillefer; Sirona Dentsply, Charlotte, USA) to the nearest millimeter from the cemento-enamel junction to the gingival margin in accordance with the Miller’s classification^29^.

#### Clinical attachment level (CAL)

CAL was measured with a graduated periodontal probe (Maillefer; Sirona Dentsply, Charlotte, USA) in millimeter from the cemento-enamel junction (or the border of a restoration) to the base of the probable pocket^4^.

#### Full mouth plaque score

A full mouth plaque score was calculated by adapting the Plaque Index by Silness and Löe (1964) to index the plaque score on every tooth. A plaque disclosing agent was utilized (2Tone Disclosing Solution; Young Dental, Earth City, USA) to visualize plaque deposits.

Index values:

- 0: No visible plaque.
- 1: Plaque disclosed on probing.
- 2: Visible plaque.
- 3: Abundant plaque^4,29^.

#### Full mouth bleeding on probing (BOP)

BOP for all teeth was calculated as a percentage of the total number of pockets presenting with bleeding on probing, for the right set of quadrants and the left set of quadrants.

#### Tooth mobility

The Miller’s criteria were used to assess tooth mobility.

Tooth mobility index:

- Degree 0: Normal physiological tooth movement of between 0.1 and 0.2 mm in a horizontal direction.
- Degree 1: Increased mobility of the tooth to at most 1 mm in a horizontal direction.
- Degree 2: Visually increased mobility of the tooth exceeding 1 mm in a horizontal direction.
- Degree 3: Severe mobility of the tooth both in a horizontal and vertical direction^4^.

The FMPE served as the clinical parameters and were recorded at the initial visit and repeated after 6 weeks^25^.

At the initial and the 6-week follow-up visit, oral hygiene instructions and education regarding the importance of adequate plaque removal were provided. Patients were shown how to brush all the teeth twice a day for two minutes with the modified Bass technique after evening flossing (Reach Expansion Floss; Johnson & Johnson, New Brunswick, USA)^30^. A soft-bristled brush (Sensodyne Multicare Soft Toothbrush; GlaxoSmithKline, Brentford, United Kingdom) with a pea-sized amount of the provided toothpaste (Rapid Care Sensodyne; GlaxoSmithKline, Brentford, United Kingdom) was used. The patients were instructed not to use any form of mouth rinse during the duration of the study, since it could influence the outcome of treatment.

#### Non-surgical periodontal therapy (NPT)

The quadrants were all treated at the same visit. Ultrasonic debridement of plaque and calculus deposits were completed with a Piezo-electric scaler (ART-PB3; BonART, New Taipei City, Taiwan) at a frequency range between 27 and 32 kHz. Mechanical subgingival debridement was performed with hand curettes and mini fives (Gracey; Hu-Friedy, Illinois, USA). All the teeth received supragingival tooth structure and root surfaces debridement. Supragingival tooth structure was polished with a polishing cup (Nu-Pro Prophy Cup; Sirona Dentsply, Charlotte, USA fitted on a slow handpiece with a medium abrasion polishing paste (Nu-Pro; Sirona Dentsply, Charlotte, USA).

#### Laser decontamination of test quadrants

The diode laser (Picasso GaAlAs; AMD Lasers, West Jordan, USA) with a wavelength of 810 ± 10 nm was used. The laser was used with a 400 µm (0.4 mm) optical fiber. The tip was cleaved and assessed under a magnifying glass at 2× magnification to ensure a square cleavage of the tip since incorrect cleavage will result in irregular laser light distribution^19^. For periodontal pocket decontamination, it is recommended that the laser tip must be initiated. This was achieved by placing the tip on articulating paper (Thick blue 127 µm; Crosstex International, Lawrenceville, USA). Initiated tips require less energy to achieve the same amount of energy delivery to the tissues than a non-initiated tip. This has the advantage of decreasing the lasing time required and the temperature, thus reducing the risk of thermal damage to the periodontal structures^11^.

Application of the laser in the quadrants receiving NPT with the laser (test quadrants) as adjunctive laser therapy and NPT alone (control quadrants): the laser tip was applied in the pocket for the extent of time that was needed to sweep the tip through the pocket. This time was maintained at 8 ± 2 s per buccal aspect of the tooth and about 8 ± 2 s per lingual/palatal aspect of the tooth. Between lasing of pockets, any accumulated debris was removed with a moistened gauze (Premium; Crosstex International, Lawrenceville, USA). The tip was held parallel to the long axis of the tooth and 1 mm coronal of the periodontal base of the pocket. In order to ensure that the laser tip is placed at the correct pocket depth, plastic capillary tips (Purple 0.36 mm tip; Ultradent, South Jordan, USA) was calibrated in millimeter segments (as found on a periodontal probe) and placed over the laser fiber and handpiece before laser decontamination commenced. The periodontal pocket depths from the FMPE parameters served as a reference guide. If a deeper pocket required a longer fiber length, then the fiber was fed through the laser handpiece.

The laser decontamination treatment commenced from the most distal tooth of the test quadrants towards the most mesial. Laser decontamination started on the buccal aspect of the tooth, from the distal aspect of the periodontal pocket to the mesial aspect of the periodontal pocket and then repeated on the lingual/palatal aspects of the teeth. The periodontal pockets of all the teeth in the test quadrants were rinsed with saline and a calibrated tip (Purple 0.36 mm tip; Ultradent, South Jordan, USA). This technique allowed the saline to rinse to the appropriate depth in the pockets, based on the FMPE. Following the lasing and saline rinse the laser decontamination was repeated for a second time for an additional 8 s at the buccal and palatal/lingual aspects of the teeth. This timeframe was chosen based on previous research findings^23^ where 10 s was shown to cause thermal damage, as well as that bacterial decontamination of more than 99% simply cannot be achieved in less than 10 s. In order to avoid any patient discomfort, treatment was completed under local anesthetic (Xylotox Plain; Adcock Ingram, Johannesburg, South Africa). Non-adrenalin local anesthesia ensured that the presence of adrenalin could not have an impact on the level of chromophores in the soft tissue of the periodontal pocket. The necessary personal protective equipment (Protective fitover goggles; AMD Lasers, West Jordan, USA) to operate the diode laser was worn by the operator and the patient^31^. The laser application in the test quadrant pockets occurred after the tip was initiated on articulation paper. A study has indicated damage to the root surface at settings exceeding 1.5 W^23^. For this split-mouth study, the power setting of 1 W^32^ at a frequency of 50 Hz^22^ was selected. The diode laser (Picasso GaAlAs; AMD Lasers, West Jordan, USA) was set at continuous wave for the “decontamination”. The spot diameter at tissue is 400 µm with the spot area at tissue 0.0013 cm^2^, providing a 796 W/cm^2^ peak power density. The energy density was 32 J/cm^2^.

### Study outcomes

The primary outcome at the end of the first visit was the establishment of the bacterial and clinical baseline parameters. This was followed by NPT alone (control side) and NPT with the laser (test side) as an adjunct according to the assignment of test and control quadrants.

The secondary outcome was achieved at the 6-week follow-up. At this visit the bacterial and clinical parameters were recorded again as per the study protocol.

### Sample size

Upon conclusion, the study population consisted of 25 patients. The statistical power of 80% with the *f* (α,β) set at 7.85^33^ was used in order to detect a significant difference of 1.0 mm for clinical attachment level (CAL) within person^23,29^. The α = 0.05 and standard deviation of the difference [SD] = 1.39 mm was determined based on the 25 patients treated. These parameters confirmed that a sample size of 16 based on the previously cited formula^33^ was statistically acceptable. Previous studies treated 22 patients^23,34^. Additionally, the split-mouth model was assessed in terms of baseline clinical and bacterial parameters and no significant difference (*P* > 0.05) was detected at the start of this study^35^.

### Randomization

The selection of the test and control quadrants were standardized for the split-mouth design. After the completion of the bacterial collection, a set of quadrants were assigned to either the “test” group (study quadrants receiving NPT with the laser as an adjunct) or the “control” group (study quadrants receiving NPT alone). The opposing quadrants were assessed as a set. Numbers were assigned to the bacterial collections at each visit, as well as to the FMPE in order to maintain the anonymity of the patients.

It has been cited that bias could be introduced in split-mouth studies upon the selection procedure for which quadrants received the adjunctive diode laser therapy^36^. In order to eliminate bias of quadrants selection, quadrants were assigned as either test or control by a process of simple randomization^37,38^. This split-mouth design ensured that the patient served as the test and the control. The randomization could therefore be performed with a simple coin toss, which resulted in a balanced allocation of test and control quadrants, each consisting of one upper and the corresponding lower quadrant. The clinician was blinded to which set of quadrants would be the test or control quadrants, as randomization was only performed after the bacterial collection of the first intervention and completion of the NPT in all four quadrants. In order to reduce the bias by patients realizing which side received the diode laser as an adjunct, the laser tip was swiped through the pockets with the same technique, but only the aiming beam (at the weakest brightness setting as per the operating instruction manual) and saline rinse. The treatment was performed by one clinician (SMVS) after calibration of the prescribed NPT by a Specialist Periodontist in the Department (HH).

The manufacturer’s instructions of the bacterial testing kit, stated that the bacterial analysis is DNA-based, no special terms of transport need to be observed (Micro-IDent-11; Hain Lifescience, Nehren, Germany). Samples may be stored in a refrigerator. In this study, the collected samples were blinded by assigning a number to each kit, refrigerated and couriered in a refrigerated cooler at ± 10 °C within 24 h of collection.

### Statistical analysis

All the bacterial and clinical data were collected and inserted into an Excel spreadsheet (Excel 2012; Microsoft, Redmond, USA) and analyzed with R Core Team (R: A language and environment for statistical computing; R Foundation for Statistical Computing, Austria, 2013).

### Data processing of bacterial and clinical parameters

The analysis report generated from the bacterial assessment kit^28^ (Micro-IDent-11; Hain Lifescience, Nehren, Germany) provide the bacterial pathogen parameters as crude “ < and > ” ranges (Fig. 1). To facilitate statistical analysis these crude ranges were converted to pathogen concentration ranges and captured as decimal values (as confirmed to be correct with the manufacturer^35^) (Table 1).

**Table 1:** Interpretation of pathogen concentration from the bacterial assessment kit (excluding *A.a.).*

KEY	Reported Pathogen concentration	Exact pathogen concentration range	Captured data for statistical analysis
−	< 10^4^	10^0^–10^3.99^	0.35
(+)	10^4^	10^4^	0.4
+	< 10^5^	10^4.01^–10^4.99^	0.45
++	< 10^6^	10^5^–10^5.99^	0.55
+++	≥ 10^6^	10^6^–10^∞^	0.6

The results in Table 2 show the pathogen concentration as per the bacterial assessment kit and the results captured to facilitate statistical analysis for *A.a.* Upon analysis the values were converted back to the range for accuracy and interpretation purposes^28,35^.

**Table 2:** Interpretation of pathogen concentration from the bacterial assessment kit for *A.a.*

KEY	Reported Pathogen concentration	Exact pathogen concentration range	Captured data for statistical analysis
–	< 10	10^0^–10^2.99^	0.25
(+)	10^3^	10^3^	0.3
+	< 10	10^3.01^–10^3.99^	0.35
++	< 10	10^4^–10^4.99^	0.45
+++	≥ 10^5^	10^5^–10^∞^	0.5

**Figure 2:**
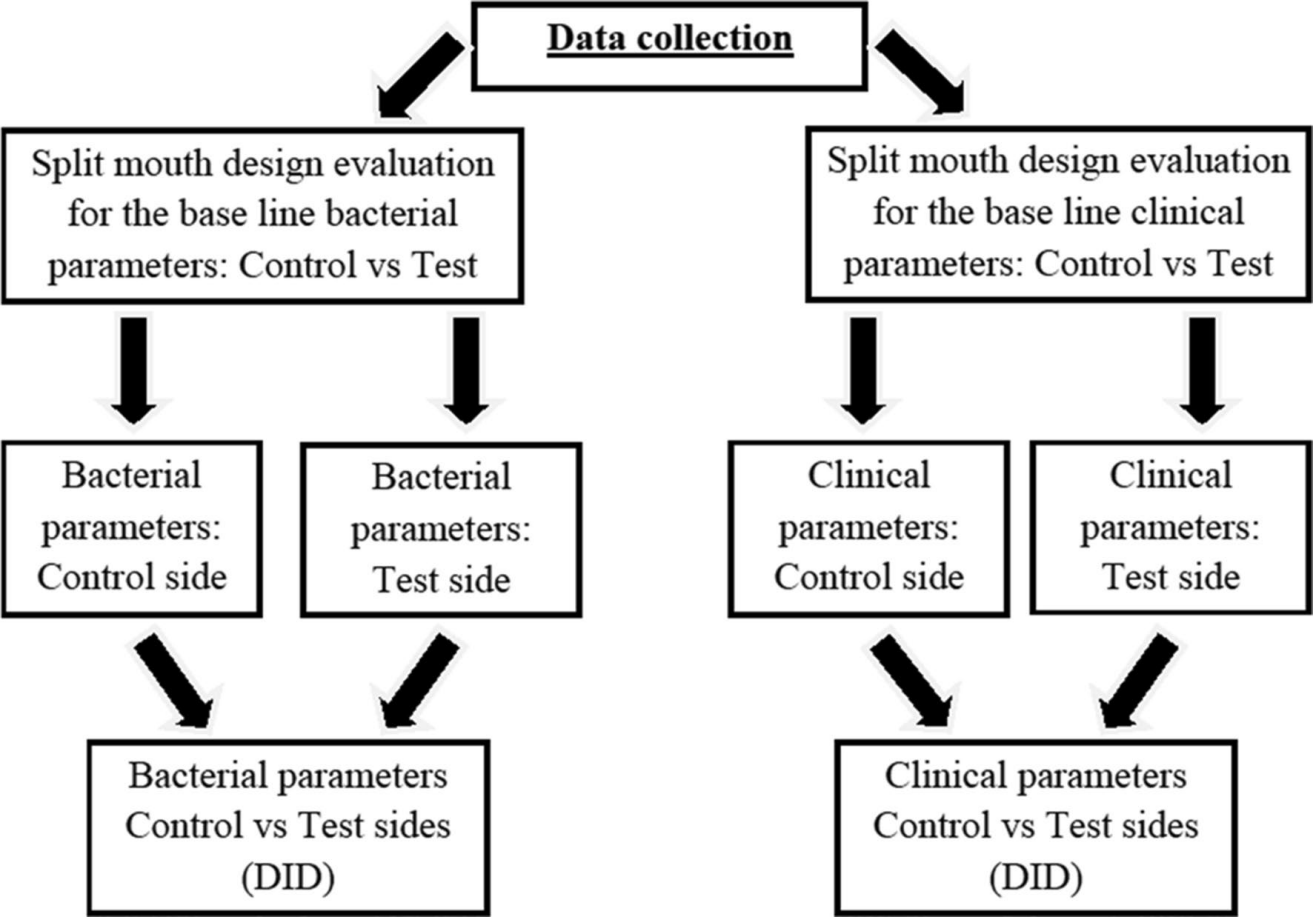
Flow diagram of the data collection and statistical analysis of all the bacterial and clinical parameters.

Each pair of quadrants (control or test) were assessed for the bacterial collection and the clinical parameters to evaluate statistical significance. *P* < 0.05 was considered statistically significant. Comparison of the FMPE for the set of the control quadrants and the test quadrants were calculated. The means and standard deviations were calculated from each clinical parameter that was recorded including the bacterial assessment from the PCR results, at the test and control sides and statistically analyzed. The statistical analysis performed was based on a one-sample *t* test. It was performed on the differences of the bacterial parameters before and after treatment. It was therefore essentially a paired *t* test of the mean values. The degrees of freedom used for statistical analysis with the *t* test was calculated as df = 24. The statistical analysis of the data considered each of the bacterial species before and after treatment. In order to determine if NPT with the laser (test side) as an adjunct to NPT alone (conventional management) made a statistical difference, the final analysis^35^ of the data was completed with the use of the “difference in differences” (DID) (Fig. 2).

The data had to be interpreted based on the mathematical values in the equation to prevent a reverse casualty. This reverse casualty could occur, since the data is multi-dimensional data that was obtained over the course of the treatment. The difference in differences (DID) was obtained when the values considered for this test was calculated in the following mathematical equation:

$${\text{DID}} = ({\text{Value}}\;{\text{before}}\;{\text{NPT}}\;{\text{with}}\;{\text{laser}} - {\text{Value}}\;{\text{after}}\;{\text{NPT}}\;{\text{with}}\;{\text{laser}}) - ({\text{Value}}\;{\text{before}}\;{\text{NPT}}\;{\text{alone}} - {\text{Value}}\;{\text{after}}\;{\text{NPT}}\;{\text{alone}}).$$

This test therefore, calculated the final outcome of the average change in the bacterial parameters of the pockets that occurred over 6 weeks for the NPT with the laser (test side) as an adjunct in relation to the NPT alone (control side).

#### Statistical analysis for the bacterial parameters before and after treatment

The statistical analysis of the bacterial parameters was performed based on a one-sample *t* test. It was performed on the differences of the bacterial parameters before and after treatment. It was therefore essentially a paired *t* test of the mean values. The statistical analysis of the data considered each of the bacterial species before treatment and after 6 weeks at a significance of *P* < 0.05.

The statistical analysis for the clinical parameters for NPT alone (control side) (before treatment and after 6 weeks) as well as for the NPT with the laser (test side) as an adjunct (before treatment and after 6 weeks) was performed in a similar manner. The statistical analysis performed was based on a one-sample *t* test. It was performed on the differences of the clinical parameters before and after treatment. The statistical analysis of the data considered each of the clinical parameters before and after treatment at a significance of *P* < 0.05.

### Ethical approval

All procedures performed in the present study involving human participants were in accordance with the ethical standards of the institutional and/or national research committee and with the 1964 Helsinki declaration and its later amendments or comparable ethical standards.

### Informed consent

Informed consent was obtained from all individual participants included in the study.

## Results

### Included study population

A total of 25 patients were present at the end of the study with 12 males and 13 females. The average age was 45 (± 6) years. The majority of the patients reported no medical conditions and five presented with controlled diabetes with hypertension. Twenty patients did not smoke and five smoked less than 10 cigarettes per day. In the control quadrants 180 single rooted and 120 multi-rooted teeth were assessed. The test quadrants presented with 173 single rooted and 125 multi-rooted teeth. None of the teeth presented with furcation lesions.

### Bacterial parameters

The data processing applied from the bacterial reports (Fig. 1) was efficient since clear parameters were established with the manufacturer (Tables 1, 2). This facilitated the statistical analysis for the results from the 25 patients, taking the decimal values into consideration once the mean values have been calculated (Table 3). Interpretation of the results represented in Table 3 using *T.d.* as an example, the control side had statistical significant bacterial parameter decreases based on the decimal values. Therefore, the control side for *T.d.* had a bacterial parameter change from 0.47 to 0.41.

**Table 3:** PCR bacterial range from testing kit applied to illustrate the precise bacterial parameter range after 6 weeks.

Bacterial species	*n* = *25*	Before treatment	After treatment	Statistical difference found with the reported PCR value from testing kit	Statistical difference found with the processed decimal value
***A.a.***					
Control side	25	0.28 (< 10^3^)	0.294 (< 10^3^)	None	None
Test side	25	0.29 (< 10^3^)	0.298 (< 10^3^)	None	None
***P.g.***					
Control side	25	0.468 (< 10^5^)	0.448 (< 10^5^)	None	None
Test side	25	0.468 (< 10^5^)	0.44 (< 10^5^)	None	None
***T.f.***					
Control side	25	0.49 (< 10^5^)	0.474 (< 10^5^)	None	None
Test side	25	0.482 (< 10^5^)	0.47 (< 10^5^)	None	None
***T.d.***					
Control side	25	0.47 (< 10^5^)	0.41 (< 10^5^)	None	Yes
Test side	25	0.4 (10^4^)	0.424 (< 10^5^)	Bacterial increase (10^4^ to 10^5^)	None
***P.i.***					
Control side	25	0.424 (< 10^5^)	0.392 (< 10^4^)	Bacterial decrease (10^5^–10^4^)	None
Test side	25	0.388 (< 10^4^)	0.394 (< 10^4^)	None	None
***P.m.***					
Control side	25	0.394 (< 10^4^)	0.366 (< 10^4^)	None	None
Test side	25	0.374 (< 10^4^)	0.382 (< 10^4^)	None	None
***F.n.***					
Control side	25	0.382 (< 10^4^)	0.51 (< 10^6^)	Bacterial increase (10^4^–10^6^)	None
Test side	25	0.51 (< 10^6^)	0.508 (< 10^6^)	None	None
***C.r.***					
Control side	25	0.508 (< 10^6^)	0.382 (< 10^4^)	Bacterial decrease (10^6^–10^4^)	None
Test side	25	0.388 (< 10^4^)	0.376 (< 10^4^)	None	None
***E.n.***					
Control side	25	0.376 (< 10^4^)	0.362 (< 10^4^)	None	None
Test side	25	0.36 (< 10^4^)	0.364 (< 10^4^)	None	None
***E.c.***					
Control side	25	0.364 (< 10^4^)	0.376 (< 10^4^)	None	None
Test side	25	0.39 (< 10^4^)	0.394 (< 10^4^)	None	None
***C.s.***					
Control side	25	0.394 (< 10^4^)	0.36 (< 10^4^)	None	Yes
Test side	25	0.364 (< 10^4^)	0.378 (< 10^4^)	None	None

Value in () as per bacterial assessment with testing kit “ < ; > ” (Micro-IDent-11; Hain Lifescience, Nehren, Germany).

The effectiveness of NPT alone (control side) on the bacterial parameters at the first intervention was compared to the bacterial parameters of the follow-up visit (6 weeks later) on the control sides. Table 4 demonstrates the difference in the mean value of the 25 patients for the bacterial collection after NPT alone was performed on the control side. The mean was calculated by subtracting the initial bacterial species parameters from the first intervention (before NPT alone commenced) from the bacterial collection at the second visit (6-week follow-up visit for bacterial collection after NPT alone of the first intervention).

**Table 4:** Difference between the first visit and the 6-week follow-up for bacterial parameters.

Bacterial parameters for NPT alone (control side)			Bacterial parameters for NPT with the laser as an adjunct (test side)	
Bacterial spp.	Mean difference in the Bacterial spp. count	*P* value	Mean difference in the Bacterial spp. count	*P* value
*A.a.*	− 0.014	0.356	− 0.008	0.356
*P.g.*	0.02	0.376	0.028	0.069
*T.f.*	0.016	0.484	0.012	0.491
*T.d.*	0.06	0.002	− 0.024	0.110
*P.i.*	0.032	0.115	− 0.006	0.704
*P.m.*	0.028	0.079	− 0.008	0.405
*F.n.*	− 0.128	5.008	0.002	0.890
*C.r.*	0.126	1.714	0.012	0.298
*E.n.*	0.014	0.183	− 0.004	0.491
*E.c.*	− 0.012	0.207	− 0.004	0.692
*C.s.*	0.034	0.005	− 0.014	0.089

Table 4 demonstrates that there was a significant reduction in the bacterial colonies of *C.s.* and *T.d*. This difference between the bacterial spp colonies for *T.d.* and *C.s.* had *P* = 0.002 and *P* = 0.005 respectively. *A.a.,* *E.c.* and *F.n.* demonstrated an increase in the bacterial spp parameter after NPT alone (control side), with no statistical significance. The effectiveness of NPT with the laser (test side) as an adjunct on the bacterial parameters at the first intervention was compared to the bacterial parameters of the follow-up visit 6 weeks later (after NPT with the laser as an adjunct). Table 5 demonstrates no significant reduction in the bacterial ssp for the NPT with the laser (test side)*.* A slight increase was demonstrated in *A.a.,* *C.s.,* *E.c.,* *E.n.,* *P.i.,* *P.m.* and *T.d.* bacterial spp on the NPT with the laser (test side), however this difference was not statistically significant. The remaining bacteria parameters demonstrated a slight decrease, which was not statistically significant.

**Table 5:** Difference in differences (DID) of bacterial parameters for test side compared to control side.

NPT with the laser as an adjunct (test side) compared to NPT alone (control side)		
Bacterial spp	DID Mean	*P* value
*A.a.*	0.006	0.733
*P.g.*	0.008	0.672
*T.f.*	− 0.004	0.852
*T.d.*	− 0.084	0.004
*P.i.*	− 0.038	0.216
*P.m.*	− 0.036	0.098
*F.n.*	0.13	3.235
*C.r.*	− 0.114	6.663
*E.n.*	− 0.018	0.185
*E.c.*	0.008	0.557
*C.s.*	− 0.048	0.004

The difference in differences (DID), calculated the final outcome of the average change in bacteria parameters that occurred over time for the NPT with the laser (test side) as an adjunct in relation to the NPT alone (control side) for the 25 patients. For example: Table 5 demonstrates *A.a*. with the DID mean (0.006) and (*P* = 0.733), illustrating no significant difference between the NPT with the laser (test side) as an adjunct nor for the reduction of *A.a.* at NPT alone (control side) (Table 5). The mean value − 0.014 (*A.a.* NPT alone) and − 0.008 (*A.a.* NPT with the laser (test side) as an adjunct; Table 4) indicates a slight increase in *A.a.* numbers. A significant difference between the control and the test side would be considered for a *P* < 0.05. Based on *P* = 0.356 (*A.a.* NPT alone) and *P* = 0.356 (*A.a.* NPT with the laser (test side) as an adjunct) it can be deduced that these methodologies are equally in-effective in reducing *A.a*. (Table 4).

Table 5 therefore demonstrates that NPT with the laser (test side) as an adjunct resulted in a statistically significant increase in the bacterial colonies of *C.s.* and *T.d.* (Table 5). The mean value 0.06 (*T.d.*) (Red complex) and 0.034 (*C.s.*) (Green complex) (NPT alone), indicated a reduction in the bacterial parameters. For the laser adjunct side − 0.024 (*T.d.*) and − 0.014 (*C.s.*) (Table 4) represented an increase in the bacterial parameters when NPT with the laser (test side) as an adjunct was performed. The remaining red complex bacteria (*P.i.,* *P.g.* and *T.f.*) demonstrated no significant difference when using the diode laser as an adjunct to NPT alone (control side) (Table 5).

### Clinical parameters

Table 6 illustrates the clinical parameter outcomes of the test and control sides. The effectiveness of NPT alone (control side) on the clinical parameters at the first intervention was compared to the clinical parameters of the follow-up visit 6 weeks later. A statistically significant reduction in the clinical parameters for PPD and CAL (*P* = 0.0002) was noted for the control side. The PI% and BOP% had a 310% and 240% reduction respectively, with no statistical significance. The effectiveness of NPT with the laser (test side) as an adjunct on the clinical parameters before treatment was compared to the clinical parameters of the follow-up visit 6 weeks later is shown in Table 6. The use of the laser as an adjunct to NPT resulted in no significant reduction in any of the clinical parameters. The PI% and BOP% each had a 320% reduction, with no statistical significance. The REC for both NPT alone (control side) and NPT with the laser (test side) had no difference in the mean, therefore the *P* value could not be calculated (Table 6).

**Table 6:** Difference between the first visit and the 6-week follow-up for clinical parameters.

Clinical parameters for NPT alone (control side)			Clinical parameters NPT with the laser as an adjunct (test side)	
Clinical parameters	Mean difference in the clinical parameters	*P* value	Mean difference in the clinical parameters	*P* value
PPD	0.4644	0.0002	0.4984	3.852
REC	0	N/A	0	N/A
CAL	0.4648	0.0002	0.5288	5.588
PI	48.4	2.2	49.4	2.2
BOP	41.64	8.038	50.144	1.302

Table 7 demonstrates the difference in differences (DID), calculating the final outcome of the average change in clinical parameters that occurred over time for NPT with the laser (test side) as an adjunct in relation to the NPT alone (control side). The test side (NPT with laser as an adjunct) compared to the control side (NPT alone) and no significant difference, with the exception of BOP% which was statistically significant *P* < 0.05 (Table 7).

**Table 7:** Difference in differences (DID) of clinical parameters for test side compared to control side.

NPT with the laser as an adjunct (test side) compared to NPT alone (control side)		
Clinical parameters	DID Mean	*P* value
PPD	0.034	0.763
REC	0	N/A
CAL	0.064	0.586
PI	1	0.245
BOP	8.504	0.005

## Discussion

The findings of this study failed to reject the null hypothesis (H_0_) for the improvement of the treatment outcomes (clinical/bacterial parameters), except BOP where the alternative hypothesis (H_a_) was accepted for NPT with laser as adjunctive therapy.

Numerous studies investigating the application of diode lasers in the management of chronic periodontitis have been published, however these studies demonstrate significant heterogeneity in their study design and have yielded varied results. It was also difficult to compare the findings of these studies, due to variations in the laser wave lengths utilized and clinical parameters assessed^23,25,34,39,40^.

The literature search identified four studies (Table 8) which evaluated the diode laser (810 ± 10 nm) as an adjunct in the management of chronic periodontitis. This study was modelled on the premise of the split-mouth design found in multiple studies with lasers as an adjunct to NPT. The methodologies vary, but in essence, multiple NPT and lasing visits were performed^22–25^. Patients with a confirmed diagnosis of chronic periodontitis and probing pocket depth (PPD) of > 4 mm were included in the studies (Table 8). Two studies utilized a split-mouth technique^23,25^ and assessed the efficacy of scaling, root planing and polishing with laser (test group) compared to scaling, root planing and polishing alone (control group). The study with 49 patients (12 in the control group and 37 in test group) was included in this literature review as a diode laser (809 nm) was used and it was one of the only two studies that assessed bacterial changes^22^. The study where the control group rinsed with hydrogen peroxide was not included in the literature review due to this chemical interaction^41^. An included study compared no treatment (control group) to non-surgical periodontal therapy, as well as non-surgical periodontal therapy with the laser^24^.

**Table 8:** Comparison of studies utilizing a 810 ± 10 nm diode laser as an adjunct in the management of chronic periodontitis.

Author	Laser	*n*	Power	Time	Technique	Improvement in clinical parameters (test vs control)	Improvement in bacterial parameters (test vs control)
Moritz et al., 1997	805 nm	*n* = 49	2.5 W, 400 µm	10 s	Pocket lased	None assessed	No statistical significance
Kreisler et al., 2005	809 nm	*n* = 22	1.0 W, 600 µm	10 s, interruption 30 s	Fiber parallel to pocket. Moved along long axis of tooth 1 mm coronal to periodontal pocket	Improvement-statistically significant for PPD, CAL. (Parameters assessed: clinical attachment loss, tooth mobility, pocket depth, plaque index, gingival index, sulcus fluid flow rate)	None assessed
Zingale et al., 2012	810 nm	*n* = 25	0.9 W, ? µm	30–45 s (cw)	Removal of pocket epithelium	Improvement—no statistical significance (Parameters assessed: bleeding on probing, clinical attachment loss, pocket depth)	None assessed
Alves et al., 2013	808 ± 5 nm	*n* = 36	1.5 W, 400 µm	20 s	Pocket lased	Improvement—no statistical significance (parameters assessed: clinical attachment loss, pocket depth, bleeding on probing, recession, plaque index)	Improvement—no statistical significance

Only two of the relevant studies (Table 8) identified and assessed the changes in bacterial parameters within the periodontal pockets. The total bacterial parameters and number of pigmented bacteria were assessed and found to be reduced, however, this reduction was not statistically significant. These studies used bacterial culturing techniques to analyze subgingival micro-organisms. Bacteria were collected with sterile paper points and cultured in petri dishes, after which colony forming units (CFU) were counted. Specific pigmented bacterial colonies were identified via gram staining. The drawback with the agar plate method for the counting of CFU is that there are bacteria that could be incorrectly identified, since they look similar on the growth medium, possibly resulting in a higher CFU identification number for that bacteria^22,25^. Molecular microbial techniques—such as PCR have become available to assess periodontal pathogens, which overcame these drawbacks^42^.

This split-mouth study utilized PCR to assess the changes in bacterial parameters. The control side demonstrated only a significant reduction in the bacterial colonies of *C.s.* (*P* = 0.005) and *T.d.* (*P* = 0.002). A slight increase in the bacterial spp of *A.a.* (0.294), *E.c.* (0.376), and *F.n.* (0.51) was demonstrated in Table 3 on the control side, however, this increase was not statistically significant. The test side demonstrated no significance for the increase of the bacterial ssp of *A.a.,* *E.c.,* *E.n.,* *C.s.,* *P.i.,* *P.m.* and *T.d.* (*P* > 0.05) in Table 4. Further analysis (performed with DID in Table 5) determined the effectiveness of the laser as an adjunct when comparing the control side to the test side for the bacterial parameters. This study demonstrated that the laser as an adjunct to NPT (test side) resulted in statistically significant increases in the bacterial colonies of *C.s.* and *T.d.* (*P* = 0.004). The finding of an increase in *C.s.* and *T.d.* in the test side of this study could not be completely clarified upon an investigation of the literature. The authors speculate that the microbial sampling technique may have only collected bacteria found within the pocket, which does not account for bacteria that have penetrated the soft tissue walls of the pocket. Thus the bacteria that were collected were possibly still in the phases of establishing their position within the pocket biofilm. *T.d.* has been demonstrated to have the necessary genes for chemotaxis and motility to effectively penetrate soft tissue. In a study comparing wild-type *T.d.* and mutated *T.d.*, it was found that the mutated *T.d.* was non-motile and unable to overcome the soft tissue barrier^43^. Thus the *T.d.* sampled from the test side upon follow-up could potentially be mutated *T.d.* with an impaired virulence. The other consideration is that although an increase in *T.d.* and *C.s.* was found, the microbial analysis does not discriminate between virulent or damaged bacteria. *C.s.* forms part of the green complex bacteria and its increase in value could be considered to be associated with a healthier biofilm complex.

With regards to the remaining red complex bacteria *P.g.* and *T.f.,* no significant difference *P* = 0.672 and *P* = 0.852 were found when comparing the laser as an adjunct to NPT alone. The results from the bacterial parameters (*A.a.,* *P.i.* and *P.g.*)^22,25^ at the same follow-up time period was similar to this study, with no significant difference demonstrated in the laser test side. It is important to note that even when present in low numbers, *P.g.* play a significant role in altering the composition of the biofilm. *P.g.* can be considered as a “keystone pathogen” as it directs the genetic response of the other organisms^44^. This could explain why the 810 nm diode laser as an adjunct did not have a significant impact on the levels of *P.g.* in this study (Tables 4, 5). The effect of the 810 nm diode laser on the periodontal pathogen *P.g.* on blood agar has been evaluated^45^. The hypothesis was that the blood agar represented the periodontal pocket since the haemoglobin will absorb the laser energy in a similar manner as the periodontal pocket. The conclusion was that the 810 nm laser results in ablation of both *P.g.* and the agar^45^. Therefore, the true ablation capacity of the diode laser for *P.g.* could be masked by the haemoglobin absorption and the resulting cumulative absorption in the periodontal pocket. This could explain the significant decrease in BOP with the laser as an adjunct in this study. Although *P.g.* was not statistically decreased, the inflamed pocket that contain the haemoglobin chromophores absorbed the laser energy, resulting in the removal of the inflamed tissue.

Most of the studies assessed the same clinical parameters, namely probing pocket depth (PPD), recession (REC), clinical attachment loss (CAL), full mouth plaque score and bleeding on probing scores (BOP) for the patients participating in the studies. Three studies assessed improvement in clinical parameters^23–25^. The results indicated no significant differences for BOP, CAL and PPD^24^ and BOP, GI and PI^23^ respectively. In this study NPT alone (control side) demonstrated reductions in BOP% in Table 6, however the results were not statistically significant. NPT alone (control side) demonstrated a statistically significant reduction in the clinical parameters for CAL and PPD at *P* = 0.0002*.* On comparison of NPT with the laser (test side) as an adjunct at the first and 6 week follow-up, BOP % was not significantly different. The NPT with the laser (test side) as an adjunct demonstrated no significant reduction in any other clinical parameters (Table 6). Further analysis was performed to determine the effectiveness of NPT alone (control side) compared to the NPT with the laser (test side) as an adjunct for the clinical parameters. This data was completed with the DID formula. The results demonstrated no significant difference between the NPT with the laser (test side) as an adjunct and NPT alone (control side) for improvement in clinical parameters, with exception of BOP% which demonstrated significant improvement on the NPT with the laser (test side) as an adjunct compared to NPT alone (control side) (Table 7).

The diode laser achieved considerable bacterial elimination from periodontal pockets^22^, with improved clinical parameters and resulted in a conclusion of the laser as a potential adjunct to conventional scaling and root planning^23^. In contrast, in other studies the diode laser was not of any additional benefit to NPT^24,25^. The clear diversity in these results and the incorrect interpretation of the bacterial analysis^22^ attributed to the heterogeneity of the study parameters, making direct comparisons or conclusions with regard to the true advantageous effect difficult^39,42^. The assessed studies (Table 8) concurred on the potential use of diode lasers as an adjunct to non-surgical periodontal therapy in the treatment of periodontitis. Further studies however were cited to be required^22,23^. In a systematic review on lasers as an adjunct, marked differences within study methodologies were found, making direct comparisons between pre-operative and post-operative treatment parameters difficult^18^. This was echoed in a meta-analysis stating that the results in the literature were conflicting due to heterogeneity between studies regarding wavelengths, fiber tip diameters, power settings, laser application times and the number of lasing sessions. Unfortunately, no meta-analysis could be performed with regard to microbiological outcomes^5^. The assessment of the evidence-based clinical practice guidelines of NPT by the American Dental Association Council on Scientific Affairs, concluded that the evidence of benefit-to-adverse effect balance is uncertain^46^. Various authors concluded that no full consensus on the efficacy of adjunctive laser therapy has been demonstrated, due to extensive heterogeneity between studies^1,8^. The American Academy of Periodontology published a best evidence consensus statement that addressed the efficacy of laser therapy used alone or as an adjunct to non-surgical and surgical treatment of periodontitis and peri-implantitis^47^. This review evaluated RCTs investigating non-surgical periodontal treatment combined with laser therapy versus laser therapy alone, in patients with moderate to severe chronic periodontitis. Current available evidence is inadequate to conclude that laser therapy alone is either superior or comparable to conventional periodontal therapy in terms of clinical improvement in probing depth and CAL in the treatment of moderate to severe chronic periodontitis. Some evidence suggests that the adjunctive use of Er:YAG or Nd:YAG lasers was beneficial as an adjunctive to conventional periodontal therapy in deep periodontal pockets (≥ 7 mm). The current evidence however fails to demonstrate a beneficial long-term effect (> 48 months) of the laser as an adjunctive therapy to non-surgical treatment of periodontitis^47^.

An essential difference in this study was that the full-mouth periodontal examination, bacterial collection, NPT and laser application was performed and assessed as a single intervention. Thus, the patient had no prior knowledge of the study and could therefore not perform an elevated level of oral hygiene practices before the first intervention. This study design was important as another study performed the baseline bacterial collection at the start of the second week, after one non-surgical periodontal therapy appointment had already been performed^22^. The studies discussed in Table 8 followed protocols that involved multiple lasing and NPT visits. Bacterial analysis and assessment of clinical parameters was also performed after multiple NPT and laser interventions were performed. The question that thus becomes apparent: “Is it the laser that results in the clinical and bacterial parameters changing, or simply the multiple non-surgical periodontal therapy visits that were performed?” Most studies have also assessed the outcomes of multiple laser interventions in combination with repeated sessions of NPT. Thus the true effect of a single diode laser (810 nm) application on the pocket biofilm has not been established. Based on the findings of this study, relating to the results after a single intervention of NPT and laser treatment, the authors suggest that the continuous disruption of the biofilm in the studies could have brought about the changes in clinical parameters observed.

## Conclusion

This study demonstrated that a single diode laser (810 nm) application, as an adjunct at the first intervention of NPT had no significant effect in the reduction of the bacterial parameters nor resulted in an overall improvement of the clinical parameters.

It is the author’s conclusion that the true effect of the diode laser on periodontal pathogens on a cellular and molecular level has yet to be established. Further studies evaluating the structural changes that could occur in the periodontal pathogens (i.e. cell wall destruction, bacterial virulence reduction, decreased colony forming ability etc.) could be an essential component to establish the true effect of the diode laser.

## Limitation

Additional 6-week follow-up intervals to assess the bacterial and clinical parameters over a 3 month period could have provided more insight into long-term effects of the study intervention.
